# How choice became a goal in itself: analysing the emphasis on choice and control at the end of life through the work of Charles Taylor

**DOI:** 10.1007/s11019-025-10321-3

**Published:** 2026-01-10

**Authors:** Els van Wijngaarden

**Affiliations:** Department of Anesthesiology, Pain and Palliative Medicine, Radboud University Medical Center, Geert Grooteplein Zuid 10, 6525 GA Nijmegen, The Netherlands

**Keywords:** Good death, Dying well, Choice, Control, Dialogical understanding, Charles Taylor

## Abstract

Choice has become a defining lens through which modern people imagine dying. A ‘good death’ is increasingly associated with the ability to articulate and realise personal preferences regarding the dying process: how, where, with whom, and even when one dies. In this paper, I examine the cultural shift that has elevated choice to a central moral ideal at the end of life. My research questions are: Why does choice matter so profoundly in contemporary societies? And how does this aspiration shape the way people live towards the end of life, and ultimately die? To answer these questions, I first offer a historical-cultural account of how choice has gradually come to function as a goal in itself. Drawing primarily on Charles Taylor’s analyses, I bring his work into dialogue with that of other scholars including Giddens, Rosa, Dodds, and Mol. I then identify three existential implications of this shift that complicate contemporary engagement with death and dying: (1) an increasingly mastery-oriented stance towards life and death, (2) the arbitrariness of meaning, and (3) an understanding of respect as unquestioning compliance with individual preferences. Finally, I propose several ways to respond to these challenges by moving beyond a narrow focus on choice and control. I argue for a rethinking of the current dominant end-of-life discourse through a more dialogical and ambivalent understanding of our relation to life and death; one that is more attuned to the lived experience and unsettling realities of dying, and better suited to enriching contemporary debates and practices surrounding the end of life.

## Introduction

*‘I want to die on my own terms’*. This phrase expresses a sentiment about dying that is commonly articulated in Western societies (Booth and Blake 2022; van Brussel 2015; van Wijngaarden & Sanders 2022; Weicht and Forchtner 2021; Young et al. 2021). It depicts a ‘good death’ as an individual, self-determined choice, in which choice is closely linked to the wish for control. That is, while the inevitability of death itself cannot be controlled, there is a growing tendency to (attempt to) control the manner, circumstances and timing of dying. If one is able to mould the dying process according to one’s preferences, it is commonly perceived as a good death (Cottrell and Duggleby 2016; Meier et al. 2016; van Wijngaarden & Sanders 2022; Zaman et al. 2021).

This perception reflects a more general distinctive feature of a prevailing contemporary Western ideal of the individual: the self is increasingly envisioned as a reflexive project for which each individual is responsible (Giddens 1991, p. 75). The modern self seeks fulfilment not only by exercising choices throughout life, but also in its final stages. Taking some form of control is perceived as a means of preserving an authentic identity to the very end (Gilleard 2022; Young et al. 2021). Moreover, acceleration of medical-technological developments has led to choice at the end of life being valued for additional reasons: older people are increasingly expressing a fear of receiving overtreatment, not wanting to live in a ‘diminished state’, and wanting to avoid suffering (Callahan and Gaylin 2017; Castelli Dransart et al. 2022; Emanuel 2014; Motamedi et al. 2021; van Wijngaarden et al. 2016). As such, the pursuit of personal choice and setting limits can also be seen (at least partly) as a self-affirmative and emancipatory response to medical power (Callahan, 1995/1988; Young et al. 2021).

This emerging emphasis on choice and control alters contemporary practices of dying: dying is increasingly conceived as a site of interventions and medical decision-making, requiring pro-active anticipation and preparation (Sallnow et al. 2022). More and more it is becoming something one ‘does’, rather than something one ‘undergoes’. This has resulted in health policies that emphasise the importance of individual choice as a component of good end-of-life care (Sutton and Coast 2012), made concrete in practices that document people’s care preferences such as living advance directives, do-not-resuscitate medallions, living wills and, in some countries, euthanasia requests. Research has shown that proactively discussing one’s options and making plans regarding the end of life does indeed (re)confirm people’s sense of agency and empowerment (Antonides & van Wijngaarden 2024; Brinkman-Stoppelenburg et al. 2014; Lemos Dekker 2021; Norwood 2009). Proactive care planning and developing meaningful advance directives help align care more closely with people’s preferences (Houben et al. 2014).

Simultaneously, however, empirical research has demonstrated that an emphasis on personal choice might partly overlook the complex and emotionally challenging nature of dying (Bandini 2020; Borgstrom 2015; Drought and Koenig 2002; Morrison 2020). In practice, it proves to be very difficult to anticipate one’s future, imagine future diseases and incapacities, and predict how one will experience these situations (Antonides & van Wijngaarden 2024; Lemos Dekker 2021; Sedini et al. 2022). Views and preferences may change over time. Moreover, one may be able to come to terms with certain disabilities in a way that was previously unforeseen and unexpected (Lemos Dekker 2021; van Wijngaarden 2024). Empirical research further shows that end-of-life care is driven less by patients’ preferences and choices than by resource constraints, physiological limits, and systemic features of healthcare practice and policy (Bailey et al. 2020; Borgstrom 2015; Borgstrom and Walter 2015; Drought and Koenig 2002).

Notwithstanding the experienced complexity, choice and control regarding the end of life remain highly valued among large parts of the general public, in healthcare systems and policies (van der Velden et al. 2018; Zaman et al. 2021). Since choice has become such an important aspiration, it is imperative to better understand its impact on how we live towards the end. To date, however, the wish for choice and control at the end of life is still under-researched, particularly in terms of the meanings attributed to this wish (Rodríguez-Prat et al. 2016; Young et al. 2021). In this article I want to address this lacuna. In order to do so, I have formulated the following research questions: why does choice at the end of life matter (so much) in our modern societies? And how does this emphasis on choice shape the way people live towards the end of life, and ultimately die?

This paper is structured as follows. First, I provide a historical-cultural account of how choice has gradually come to function as a goal in itself. Next, I identify three major existential implications of this shift. Finally, I outline several ways of approaching the end of life beyond a narrow focus on choice and control, and show how this can enrich our understanding of living towards the end of life and dying. For the theoretical framework, I draw primarily on the work of Charles Taylor, whose analysis of modernity provides a crucial lens for understanding how choice has come to occupy such a central place in our self-understanding. His accounts illuminate how modernity has reshaped morality and identity, and how these transformations manifest in everyday life (Taylor 1989, 1992, 2004, 2007). I place Taylor’s work in dialogue with that of other scholars, including two sociologists, Anthony Giddens and Hartmut Rosa, and two philosophers, Susan Dodds and Annemarie Mol. Giddens’ work helps to illustrate how the drive for control and choice is linked with the radical doubts, insecurities, and anxieties evoked by modernity’s freedom (Giddens 1991). Rosa’s analyses of ‘resonance’ (Rosa 2019) and the ‘uncontrollability of the world’ (Rosa 2020) further show how an overemphasis on choice and control may culminate in what he terms a ‘mute’ relation to the social world. Complementary research by Dodds (2000) and Mol (2008) demonstrates that reducing autonomy to choice and control in healthcare contexts obscures the relational, emotional, and material conditions in which decisions are made, and that choice attains ethical significance only when embedded in relationships that are responsive to human vulnerability.

## How and why did choice become such an important value?

To understand why choice, also at the end of life, is so prominent today, it is essential to first delve into its historical origins. For this purpose, I primarily draw on the work of Taylor (Taylor 1989, 1992, 2004, 2007). His extensive historical analyses chart the pivotal cultural shift from a premodern to a modern worldview, the latter emerging during the Enlightenment period. While this broad, mainstream perspective inevitably involves some generalisations – for instance, it does not account for the varying influences of different cultural and religious traditions within the Western world – it is indispensable for illuminating the major transitions in Western thought over the centuries, which aligns precisely with the scope of this article.

### The transition towards modernity

As Taylor describes, in premodern societies, life was seen as part of a sacred, transcendent order, with a metaphysical entity having a significance of its own, independent of how people interpreted or approached it. This all-encompassing framework gave premodern human beings a place in the world and, within that world, connected them with other people (Groot and Vanheeswijck 2018, p. 48). People would thus see themselves as part of a larger order, a link in the great chain of being (Rosa 2019, pp. 33–34; Taylor 1992, p. 3). People were imbued with a strong sense of the primacy of the group: the most important actions were those undertaken by (or on behalf of) the group. The premodern self existed only by virtue of the group (Taylor 2007, pp. 148–149). As Taylor puts it: “From the standpoint of the individual’s sense of self, it means the inability to imagine oneself outside a certain matrix” (Taylor 2004, p. 55).

This unifying metaphysical structure – consisting of shared values, collective language, traditions and modes of ritual action – provided a sense of belonging, stability, and meaning. Individuals understood themselves as deeply connected to others and to a collective framework that transcended individual interests, uniting them in purpose and identity (Rosa 2019, pp. 33, 34; Taylor 2004, pp. 51–56). Drawing on the work of Augustine, Descartes, Locke and Montaigne, among others, Taylor traces a historical evolution from these collective values to an increasingly individualistic and introspective sense of self.

For instance, Taylor has demonstrated how the theologian and philosopher Augustine (354–430) in particular already laid a crucial foundation for the modern idea of the ‘inwardness’. Although Augustine still holds the idea of God as the ultimate source of a meaningful order and knowledge (Taylor 1989, pp. 127–129), for him, God was no longer just transcendental, the light ‘out there’. Instead, God was also seen as an ‘inner’ light. With this idea, Augustine fundamentally changed the direction of the human gaze and introduced the ‘inner path’ as a way to gain insight into the truth and the rational and moral order (Taylor 1989, p. 129).

Whereas Descartes (1596–1650), an early Enlightenment thinker, built on several Augustinian ideas, such as the importance of the turn to the self and radical reflectivity (Taylor 1989, pp. 143–158), he simultaneously profoundly twisted the idea of inwardness by locating the moral sources within the human being (Taylor 1989, p. 143). For Descartes, the good or the truth was no longer a transcendent, given order that we need to discover, but rather something we have to construct by the power of thought (Taylor 1989, p. 147). This led to the Cartesian idea of the ‘hegemony of reason’ (Taylor 1989, p. 147), which refers to the power to objectify body, world and passions, and take an instrumental stance towards them (Taylor 1989, p. 147; 1992, pp. 93–103). Dismissing the idea of an enchanted world, we should consider the cosmos and the body as functional mechanisms with causal connections, and as such, domains of rational control.

Departing from the Cartesian model of rational control, Locke (1632–1704), a philosopher and medical researcher, advanced the shift by emphasising self-responsibility and independence (Taylor 1989, p. 167). He proposed that the self should be regarded as an agent capable of gaining control through a detached, objective stance towards both the world and oneself. By adopting a disciplined, instrumental approach to personal traits, habits, desires, inclinations, and emotions, individuals were thought capable of self-improvement (Taylor 1989, p. 159). Locke subsequently introduced the idea of ‘self-remaking’ or ‘re-creation’ of the self (Taylor 1989, pp. 170–171), encouraging individuals to become active builders of their own nature.

With the rise of Romanticism and the philosophies of Montaigne, Rousseau, Herder, and others, modern culture –initially focused on detached, instrumental reason and individualism– underwent what is referred to as the ‘subjective’ or ‘expressive turn’. This change introduced a new form of inwardness. The late Renaissance philosopher Montaigne (1533–1592), for instance, is considered one of the earliest thinkers on personal authenticity (Taylor 1989, pp. 178–181). While he drew on the idea of radical reflexivity, he framed it differently: rather than objectifying our nature as a tool for moral perfection, Montaigne’s ideal was ‘to come to accept what we are’ (Taylor 1989, p. 181). In this view, self-knowledge no longer meant attaining universal, impersonal insights into human nature; instead, it became a deeply individual journey, laying the foundation for a form of modern individualism that celebrates each person’s unique identity.

The works of Rousseau (1712–1778) and Herder (1744–1803) further advanced this shift in thought, culminating in an expressive vision of human life (Taylor 1989, pp. 368–376; 1992, pp. 25–29). Concepts such as ‘significance comes from within’ and ‘access to one’s inner voice’ have gained prominence (Taylor 1989, pp. 369–370). Ethics and aesthetics came to be seen as more interconnected, with the question of the good life increasingly framed in terms of sentiments and feelings (Taylor 1989, pp. 372–373). Each individual was understood as unique and original, with originality as a defining characteristic of how one ought to live. Human beings were seen as having an obligation to realise their own original self (Taylor 1989, p. 375). Over time, expressive individuation has become one of the cornerstones of modern culture.

In sum, Taylor has demonstrated how the Enlightenment, with its emphasis on increasing scientific knowledge, led to the search for rational *control* over the self, the world, and the environment (Taylor 1989, pp. 149, 175; 2007, p. 476). Furthermore, Romanticism, with its focus on expressivism and authenticity, has been a driving force behind the emphasis on personal *choice* (Taylor 1989, pp. 178–182, 390). These transitions have profoundly shaped the modern sense of self, notions of community, and moral understanding. I will briefly explain each of these below.

### The modern self-image

As pointed out above, the human self-understanding has undergone a radical change over the centuries. The modern self is viewed as an autonomous, rational, and disciplined person, able to adopt a detached stance towards the external world, relying almost exclusively on self-control and self-determination (Rosa 2019, p. 33). Furthermore, the concept of authenticity, understood as merely self-referential, has gained significant influence. We have come to think of ourselves as beings ‘with an inner voice that tells us what is the right thing to do’ (Taylor 1992, p. 26). We are called upon to be in contact with our own aspirations and desires (Taylor 1992, pp. 27–29), develop our own moral stance and define for ourselves what we consider good (Rosa 2019, p. 18; Taylor 1992, p. 81). Accordingly, we are encouraged to reflexively shape our lifestyles, sexuality, and family structures while crafting our personal biographies from countless possibilities (Giddens 1991, p. 80). As a result, the act of choosing has become a defining feature of our age, not only in terms of behaviour: ‘What should I do?’ but also in relation to personal identity: ‘Who should I be?’ (Giddens 1991), affirming the moral idea that each person has a unique way of being human. As Giddens succinctly stated: ‘We have no choice but to choose how to be and how to act’ (Giddens 1994, p. 56). Through continuous reflection and choice-making, as a day-to-day activity, we should safeguard our authenticity, resist pressures towards conformity, and as such, live our lives in our own way, and not be an imitation of anyone else.

### The modern community

Accelerated by processes such as urbanisation, industrialisation and globalisation, traditional communities have been fragmented, described as an ‘emancipation movement of the individual’ (Groot, 2016/2007, p. 24; Taylor 1989, p. 432). This has led to an erosion of social ties and disintegration of collective meaning-making practices (Giddens 1991; Taylor 1989, p. 648; 2007, pp. 146–158, 443). While these changes have prompted a search for other forms of community and cooperation (Taylor 1989, p. 649; 2007, p. 447), the new structures differ fundamentally from traditional ones: they are no longer intended to be settled for life. Indeed, relationships have also become increasingly reflexive. Commitments are now deliberate choices, valued for mutual satisfaction rather than imposed by external obligations. This transition fosters autonomy but also complicates the ability to sustain deep, enduring connections. Hence, modern communities and relationships are, in principle, temporary. They are created by individuals who connect voluntarily, based on reciprocal utility, and remain connected only as long as it serves their personal goals (Taylor 1992, p. 43). As a result, stable, embedded community life has been largely replaced by dynamic, fluid, and revocable connections, often formed for specific purposes (Taylor 1989, p. 502; 1992, p. 43).

### The modern morality

According to Taylor, modern morality is shaped by two powerful and interrelated moral developments. The first is a radical Enlightenment ethics, grounded in utilitarian thinking, which elevates human well-being, security, prosperity, and the alleviation of suffering to central social and political goals (Rosa 2019, p. 23; Taylor 1989, pp. 211, 215, 327; 2004, pp. 19–22). This shift led to the unprecedented valuation of ordinary life and a vision of society as a cooperative economic system in which rational agents work for mutual benefit (Taylor 2007, pp. 129, 171). Consequently, for the first time in human history, the enhancement of the human condition –and the reduction or elimination of suffering– became a core target of collective action and state policy (Taylor 1989, pp. 12, 331, 394; 1992, p. 104).

In response to utilitarianism, Taylor identifies a second moral turn: the rise of expressive individualism. Here, the authentic self becomes the foundational moral source. Individuals are not only autonomous agents capable of choosing their own life plans; they are also expected to live in ways that express their uniqueness and inner convictions (Rosa 2019, p. 20; Taylor 1992, pp. 28, 29, 81) (Taylor 1989, p. 375). This ethic of originality spreads across several domains of life –from work to relationships, and beliefs regarding death and dying– fuelling a moral pluralism in which norms are matters of personal preference and meaning exists within ‘a field of alternatives’ (Taylor 1989, p. 317) (Taylor 2007, p. 299). As a result, modern beliefs and ideas often have a tentative quality (Taylor 1989, p. 10), contributing to a widespread ethical uncertainty (Rosa 2019, p. 22; Taylor 1989, p. 317). That is, the question of how we should live, or die, has no fixed answer (Rosa, RES, 33). Without shared transcendental or communal anchors, individuals bear full responsibility for defining and justifying their own choices (Giddens 1991; Rosa 2019, p. 20; 2020, p. 112; Taylor 1992, p. 81).

Taken together, modern people have come to understand themselves as equal, self-responsible agents with a unique inner self, living in a world devoid of transcendental givens (Rosa 2019, p. 18; Taylor 2004, pp. 19–22; 2007, pp. 164–165). Moral sources are located within the human being. Communities are seen as dynamic and instrumental connections: they serve specific (personal) goals, often for a demarcated period. In principle, everyone is considered to be free to determine their own conception of a good life, or a good death, without any unwanted interference from others. Hence, freedom of choice itself has become a supreme value (Taylor 1992, pp. 22, 69).

## The existential challenges associated with the emphasis on control and choice at the end of life

Without trivialising its gains, it is important to recognise that modernity has also introduced substantial existential costs that strain, or even unsettle, our capacity to meaningfully engage with suffering, illness, and dying. Below, I will address three significant challenges that emerged from my analysis of Taylor’s work, namely: (1) an increasingly mastery-oriented relationship to life and death, (2) the arbitrariness of meaning, and (3) a conception of respect understood as unquestioning compliance.

### An increasingly mastery-oriented relationship to life and death

Modern societies are driven by an imperative to expand (rational) control over the world (Rosa 2019, 2020; Taylor 1989, 2007). Through the deepening of scientific knowledge and the advancement of technological capacities, humanity has aimed to master its environment, producing undeniable progress in science, technology, economics, increased prosperity and health (including longevity). Yet this rational, interventionist stance comes at a price: sustaining the status quo paradoxically requires continuous growth, acceleration, and innovation (Rosa 2019, pp. 307–310). Rosa states that this dynamic has fostered an ‘aggressive-distancing relationship with the world’, in which the world is no longer encountered as responsive or meaningful, but merely as an object to be controlled, thereby reinforcing a vicious circle (Rosa 2020, pp. 11–13). Rosa proposes that relentlessly expanding humanity’s reach has become a driver in almost all areas of life, and across all ages, from (pre-)birth till death (Rosa 2020, pp. 60–85).

The mastering attitude promises a better life. However, faced with technological systems, accelerated social processes, and an overload of life choices and perspectives, many individuals experience a sense of powerlessness and disorientation instead. As it turns out, there is a substantial gap between what Rosa terms ‘theoretical control’ and ‘actual, experienced control’ (Rosa 2020, p. 110). While people strive to achieve mastery through rational planning and choice, the contemporary world has become increasingly unmanageable, unpredictable, and complex (Rosa 2020, p. 110). This gap (between theoretical and actual control) is also clearly reflected in our contemporary relationship with death. There is a tendency to frame illness, suffering, and even dying as medical-technical problems to be solved or at least controlled (Bandini 2020; Taylor 2007, pp. 318–319). Accordingly, biomedical knowledge –grounded in physiology, diagnostics, and statistical probabilities, and expressed through laboratory data, imaging, and numerical indicators such as pain scores– has become the dominant lens through which illness and dying are perceived. While indispensable for diagnosis and treatment, this form of knowledge may eclipse experiential understandings of the body, which, in turn, could contribute to the (over)medicalisation of the bodily experience. In end-of-life care, for instance, patients’ accounts of distress, fear, or a sense of ‘being done’ tend to carry less weight in guiding clinical decision-making than measurable clinical parameters, which leaves the lived experience of dying susceptible to being undervalued or dismissed. Hence, this dominant mode of scientific biomedical reasoning may reinforce a sense of estrangement from one’s embodied existence (Bandini 2020; Rosa 2020, p. 113; Taylor 1992, p. 6; 2007, pp. 740–741).

Mol (2008) ties this mastering attitude to what she has termed ‘the logic of choice’. Within this logic, illness is treated as a disruptive exception; something external to the person that must be mastered or eliminated. In this logic, decision-making is perceived as a defining marker of agency, positioning individuals as autonomous and rational selectors of treatment options. Patients, including those nearing death, are provided with information that is grounded in scientific knowledge, and that is supposed to offer a greater sense of direction and certainty about their trajectory. They are expected to calculate the advantages and disadvantages, weigh arguments and thereby maintain some control over their trajectory (Borgstrom and Walter 2015; Ermers 2025; Sutton and Coast 2012), manifesting itself in, for example, the formalisation of end-of-life care planning. However, as noted in the introduction, when death actually approaches, the ethical and existential challenges people have to live through often prove to be far more complex and layered than anticipated (Antonides & van Wijngaarden 2024; Bandini 2020; Drought and Koenig 2002; Ermers 2025; Lemos Dekker 2021).

In practice, it turns out to be extraordinarily difficult to assess the pros and cons of an unknown future; people are frequently too frightened, too confused, or too ill. They may lack the expertise needed to make informed decisions (Mol 2008, p. 6). The often incorrect underlying assumption is that medical technology and treatment can deliver reliable prognoses and outcomes. Moreover, not all options are equally accessible. This can create the illusion of choice that obscures structural constraints and puts some people in an even more precarious and unstable situation. For instance, socio-economic and cultural dimensions –such as class, gender and ethnicity– significantly shape end-of-life experiences and decision-making processes. These factors play a significant role in determining the care or support people receive in the final phase of their lives (Elk et al. 2018; Knaul et al. 2018; Marmot 2005). By generalising choice, we may overlook how illness, uncertainties and/or limited (material) resources may undermine the very capacities presupposed by the ideal of choice. Once framed as an option, the direction of treatment becomes a private responsibility rather than a subject of collective scrutiny (Mol 2008), thereby leaving individuals to make their own decisions, and be held personally accountable for ‘mastering’ their (end-of-) life trajectory.

### The arbitrariness of meaning

A second characteristic of modernity that I want to elaborate on is that individuals are increasingly tasked with addressing existential questions and sustaining meaning in predominantly self-referential ways. Within this modern paradigm, meaning is no longer taken for granted. This shift has profound consequences: the question of meaning becomes pervasive, compelling individuals to seek, construct, and attribute value to the world independently (Taylor 1989, pp. 10–11, 178). Taylor underscores the existential burden of such freedom, noting that ‘free choice, based on nothing, contains a great emptiness in which ultimately nothing really matters anymore’ (Groot, 2016/2007, p. 18; Groot and Vanheeswijck 2018, pp. 50–51). When choices lack inherent value, the meaning assigned to them becomes somewhat arbitrary. Moreover, it becomes more or less challenging to comprehend and justify why some things are more important than others: meaning appears to be interchangeable, making difference ultimately insignificant (Taylor 1992, p. 38), and potentially fostering a form of moral relativism (Taylor 1992, p. 14). Furthermore, in a cultural context that isincreasingly deprived of collective symbolic and existential resources for interpreting (the meaning of) suffering, individuals may struggle to make sense of such unsettling experiences that remain profoundly human and universal (Illich 2003). Although these existential challenges persist, many people may lack a shared language necessary for meaningful communicating about such concerns, thus hindering the ability to deal with, let alone reconcile with, fragility and mortality (Conrad 2007; Illich 1995; Moyse 2022).

New developments, as reflected in a wide range of new rituals, personalised funerals, life-story books, and customised memorials, show that people increasingly seek their own ways of dealing with end-of-life issues. (Venbrux et al. 2009; Wouters 2002). Such practices allow individuals to express their authenticity and prepare their farewell in deeply personal ways. However, as Taylor points out, this freedom also constitutes a burden: each person must continually determine how to give shape, language, and meaning to major life events. In moments of profound existential crisis, one can rely less on established, comforting rituals and shared cultural vocabularies. As a result, at such decisive moments, individuals often find themselves having to depend on their own capacities. Accordingly, approaching the end of life has increasingly become a demanding personal undertaking; and it is up to each individual to imbue it with meaning. This loss of stable meaning structures and the resulting arbitrariness, as Giddens (1991) observes, has contributed to a pervasive sense of existential uncertainty, potentially generating serious doubt, anxiety, and insecurity.

### Respect as unquestioning compliance

A third challenge that I want to mention here is that modern ideas about respect for autonomy, personal choice and the privatisation of the good life have complicated constructive dialogue about fundamental life choices, including those related to the end of life (Antonides et al. forthcoming). In many Western countries, the question of what constitutes a good life, and thus also a ‘good death’, has increasingly been framed in terms of individual self-determination (van Brussel 2015; van Wijngaarden & Sanders 2022; Young et al. 2021). As a result, contemporary individuals are often reluctant to make substantive moral judgments about what a good life or good death might entail for others (Groot and Vanheeswijck 2018, p. 46). Normative statements are often dismissed as patronising, and what ultimately matters is commonly conceived as what each individual considers dignified or worthwhile (Mol 2008). Accordingly, appealing to individual choice often functions as a rhetorical endpoint: once the term ‘choice’ is invoked, the discussion tends to close off. Preferences are primarily understood as private matters rather than as issues for communal deliberation (Mol 2008).

This strong emphasis on personal choice and the principle of non-interference risks reducing the concept of respect to a form of unexamined acquiescence; a passive, uncritical acceptance of others’ decisions. Disagreement or questioning is easily perceived as inappropriate: *“It’s her choice*,* after all”* (Antonides et al. forthcoming). However, such a stance may inhibit open conversations and deprive individuals of opportunities to express feelings, voice concerns or raise critical questions. Empirical research on end-of-life decision-making confirms that this tendency can result in unreflective consent or even emotional disengagement, narrowing the space for meaningful dialogue among those involved in end-of-life trajectories (Antonides et al. forthcoming; Castelli Dransart et al. 2022).

Rosa’s metaphor of ‘resonance’ offers a valuable conceptual lens to better understand the implications for how we live with –and speak about– intricate end-of-life issues. Resonance, for Rosa, refers to a responsive, dynamic relationship between the self, others, and the world. This relationship is characterized by mutual exchange and openness to being affected and transformed (Rosa 2019, pp. 164–174; 2020, p. 42). Resonance may emerge in encounters with any entity, be it people, nature, music or texts, that exists beyond our control yet appeals to us and holds meaning. Resonance is not mere harmony or agreement. In Rosa’s view, resonant relationships are characterised by this element of uncontrollability (Rosa 2019, 2020, p. 43), or at least semi-controllability. That is, resonance depends on another’s ability to resist or disagree (Rosa 2020, p. 43). Only counterparts that ‘speak with their own voice’ can foster true resonance (Rosa 2019, p. 167; 2020, p. 47). Resonance thus requires a willingness to tolerate friction; these elements are essential for deeper relational connection.

In modern societies, resonance is often replaced by more instrumental relational patterns. Nature is increasingly framed as a set of resources, while interpersonal relationships tend to be approached in more transactional terms. Others may be valued primarily in relation to their contribution to individual, self-determined goals. However, Rosa suggests that when attention is primarily directed inward, we may end up hearing just ‘our own voice.’ (Rosa 2019, p. 166). In contrast, true resonance cannot occur in relationships devoid of challenge or contradiction. Respect that avoids disagreement or critique devolves into what Rosa calls ‘an echo relationship’; a self-affirming dynamic that lacks depth and responsiveness (Rosa 2020, p. 43). Applied to end-of-life conversations, this means that when respect is narrowly equated with non-interference, this can render it synonymous with unquestioned compliance. Moreover, reluctance to confront or challenge others’ decisions, out of fear of violating autonomy, can suppress precisely the kinds of deep, open-ended conversations that help individuals cope with existential hardship. A more resonant form of respect would not preclude critique or opposing views but would engage with it as part of a shared search for meaning and understanding.

## Towards a more dialogical and ambivalent understanding of end-of-life challenges

The contemporary focus on individual choice and the pursuit of control is commonly associated with freedom and self-actualisation, developments generally regarded as desirable. After all, who wants to be subjected to paternalism? As demonstrated in the previous section, this modern emphasis is accompanied by serious drawbacks that complicate our ability to meaningfully engage with the fragility of life. This section examines how these challenges might be addressed. Following Taylor (1992), Dodds (2000), and Mol (2008), my concern is not with the rejection of autonomy or freedom of choice as moral ideals, but with the detached, individualistic mode in which these ideals are regularly articulated in Western healthcare policies and practices. The issue is not choice per se, but its absolutisation: the assumption that illness and dying are best approached through individual decision-making. In practice, this focus neither expands the space people need to engage with the existential realities of dying nor delivers the promised freedom or improved care (Borgstrom 2015; Drought and Koenig 2002; Sutton and Coast 2012). Rather, it restructures everyday practices and expectations, shaping how we live towards death in ways that often do not align with the complexities of end-of-life trajectories. This section therefore suggests ways of rethinking the dominant discourse of choice and control by foregrounding a more dialogical and ambivalent understanding of our relation to life and death. This understanding is more closely attuned to lived experience and the unsettling realities of dying, as well as more conducive to enriching contemporary discussions and practices surrounding the end of life.

### Dialogical horizons of significance

Care ethical scholarship provides a fruitful avenue for rethinking the dominant paradigms of choice, control, and the autonomous, unified self. Drawing on feminist sensibilities, care ethicists have approached care for decades not as a private sentiment but as a socially and politically mediated practice through which people continually attune to themselves, others, and their environments in order to ‘maintain, continue, and repair our world so that we can live in it as well as possible’ (Tronto 1993). Rather than grounding moral life in abstract principles or in individual capacities to make rational, self-determined choices, care ethics holds that the basic conditions of human existence –relationality, interdependence, and vulnerability– carry intrinsic moral significance (Mackenzie and Stoljar 2000). Human beings are born in states of dependency and remain so throughout life. Autonomy, therefore, cannot be equated with self-sufficiency, but is viewed as a process of self-realisation mediated by reciprocity, recognition, and intersubjective relations. Moreover, in line with Taylor (1989), care ethicists challenge the presumed incompatibility of autonomy and dependency. They argue instead for a relational conception of autonomy in which networks of social interdependencies do not constrain freedom, but create the very conditions under which meaningful choice and action become possible.

In a similar vein, and with the same commitment to relationality, interdependence, and dialogue, Taylor advances the notion of ‘situated freedom’ which accounts for humanity’s rootedness in tradition and society (Groot and Vanheeswijck 2018, p. 39). It concerns a freedom that acknowledges that humans are always embedded within a preexisting world, rooted in a context they did not create nor fully master, but which nonetheless significantly shapes who they are, and who they may become. A so-called ‘horizon of significance’ is necessary for human beings to define themselves, and thus to be authentic. It establishes a foundation for collective meaning, grounded in dialogue, community and transcendence (Taylor 1992, pp. 52–53). In Taylor’s line of reasoning, one can be authentic while being inspired by sources outside oneself (Taylor 1992, p. 82). In most, if not all cases, we are unable to develop our own opinions and outlook on things through pure solitary reflection. Especially in relation to fundamental life issues, such as questions of identity and our stance towards death and dying, we formulate our views in conversation with others and society (Taylor 1992, p. 33).

Taylor thus proposes a philosophical anthropology that defines humans as fundamentally social beings who exist within, and are shaped by, ‘networks of dialogue’ (Taylor 1989, pp. 34–37). A proper understanding of identity thus requires recognition of the profound impact of family, intimate relationships, the broader social spaces humans inhabit, and the geography of social positions and functions people occupy (or are excluded from). Humans become a self only in relation with these ‘dialogical partners’; our thinking and being are always co-mediated by others. Taylor refers to this process as the ‘genealogy’ that underpins human identity and morality (Taylor 1989, p. 37). It is only through an awareness of the formative life trajectory that lies behind us that we can meaningfully reflect on our current and future selves (Taylor 1989, pp. 46–48). Authenticity and meaning thus presuppose a form of transcendence, –not necessarily in spiritual or hierarchical terms but in a secular or horizontally oriented sense– that is, the context beyond the self (Taylor 1992, p. 41). A Taylorian conception of authenticity can thus be characterised as ‘self-definition in dialogue’ (Taylor 1992, p. 66). That is, authenticity encompasses not only a mode of existence appropriated as our own, but also an acknowledgment of the horizons of significance that are already out there.

### Engaged dialogical exchange

The dialogical notions of autonomy and authenticity outlined above entail that respect for autonomy and personal choice cannot be reduced to a minimal conception of non-interference, that is, merely stepping back and leaving individuals to make decisions in near-isolation. Instead, genuine respect for autonomy requires active engagement through dialogue, grounded in sincere reciprocity between those involved (Wardrope 2015, 2016). It is important not to underestimate the challenges such existential conversations entail. Certainly, those involved (such as relatives or health professionals) should exercise caution to avoid acting in a paternalistic or intrusive manner. However, as Rosa (2019) and others have argued, autonomy and authenticity are not compromised by critical, engaged questioning and the exchange of perspectives.

Hence, engaging in dialogue around complex end-of-life issues means recognising the plurality of values and perspectives, including diverse views on what constitutes ‘dying well’ in a given case. It involves genuinely listening to others, while also sharing one’s own view. When all parties adopt an open and respectful stance, the interaction becomes a space for critical exchange, where beliefs may be deepened, advice offered, disagreements voiced constructively, and sometimes even mutual persuasion may be attempted. Such acts are not threats to autonomy; instead, they are best seen as vital expressions of meaningful respect for it. Rather than treating end-of-life choices solely as private matters of individual will, a dialogical approach highlights the importance of shared understanding, connection, recognition, and collective responsibility. This does not negate autonomy but rather situates it within a web of social relationships in which meaning is co-constructed.

### Inherent and contextual vulnerabilities

By directing attention to both inherent bodily vulnerabilities and dependencies, and the external contextual vulnerabilities imposed on people, Dodds (2000) and Mol (2008) introduce a further crucial dimension to a comprehensive understanding of our relationship to life and death. Departing from the fragility of life, Mol’s care-oriented perspective foregrounds attentiveness to the ongoing interplay between the vulnerable, mortal body and its intricate surroundings (Mol 2008, pp. 31, 34). It recognises the multiplicity and unruliness of medical possibilities, acknowledging that technologies often produce unforeseen effects (Mol 2008, p. 50). Within a logic of care, responsibility is iterative and shared rather than the property of isolated decision-makers. Instead of asking who is capable of choosing, a logic of care examines how situations of choice are produced, what conditions shape them, and how good care can be enacted through negotiation, attunement, and continuous adjustment. Along the same lines, Dodds (2000) proposes for a relational model of autonomy that takes seriously the effects of power dynamics, institutional structures, psychosocial pressures, and issues related to inequities and inequalities in health care. The model highlights that autonomy is not an isolated capacity, but one that depends on supportive social conditions. People can only deliberate, assess, and act when they are embedded in environments that nurture these capacities. For this reason, Dodds maintains that attending to human needs –those without which life is seriously harmed or diminished– is a matter of collective concern rather than a private responsibility.

The dying process and the task of assigning meaning to these difficult life events implies that meaning does not emerge from the individual’s preferences in isolation –*what do you want*,* what are your wishes?*– but from shared horizons of significance. Meaningful end-of-life conversations should therefore acknowledge that patients often discover what they value through the very act of speaking with trusted others, including health professionals. The aim is not to arrive at a definitive and fully informed answer, but to accompany the patient in articulating a stance towards dying that resonates with their lived biography. Rather than centring the discussion around autonomous decision-making and informational guidance, such conversations would attempt to create space for patients to explore how family, relationships, cultural traditions, and past experiences have shaped their identity over time, including their hopes, fears, and values regarding dying. This shifts the clinical focus from soliciting individual preferences to co-creating understanding, situating choices within a narrative of meaning. It invites health professionals to ask questions that open up the patient’s broader world: *What matters to you? Who are the people and places that have shaped you? How would you like your relationships to inform how you live towards death?*

Attending to the end of life also requires recognising inherent bodily vulnerabilities and dependencies, as well as the external and contextual vulnerabilities imposed on people. This implies inquiring what forms of care remain adequate as the body becomes increasingly fragile: *What forms of support are possible*,* given this body and its dependencies?* They should attend to how symptoms, fatigue, breathlessness, and uncertainty continually reshape what is meaningful, bearable, or desirable, allowing decisions to evolve over time. Similarly, conversations preferably also identify potential external pressures that shape a patient’s agency, such as economic circumstances, financial constraints, housing stability, family conflict, cultural expectations, institutional constraints, or unequal access to care or other support networks. Rather than presuming an autonomous chooser, end-of-life dialogues should allow for examination of the forces confronting patients and how these may constrain or distort what appears as a ‘choice’. This includes inviting patients to articulate how their relationships shape their desires for treatment, comfort, legacy, or the withdrawal of interventions, rather than pressuring them to produce a private answer detached from these relations. End-of-life decision-making is therefore iterative and should remain principally open to revision as bodies, emotions, and social conditions change. As such, health professionals can cultivate opportunities for ongoing dialogue, attuned to the shifting values and needs that accompany dying, and in this way help patients and their relatives to orient themselves towards horizons of significance in the face of imminent death.

### An ambivalent imagination

To attune our understanding of living towards the end of life more closely to the realities of contemporary dying, a final point should be made. As outlined above with reference to Rosa’s work (Rosa 2019, 2020), the prevailing focus on rational control has made it increasingly difficult to engage with the ambivalent, contingent and often unsettling dimensions of dying, as this focus tends to sustain the illusion that dying can be managed or even mastered through scientific knowledge. While acknowledging the profound benefits of medical progress, it is equally important to recognise that end-of-life treatment and care have frequently developed into almost uncontrollable technological processes; processes that do not necessarily serve the well-being of patients (Bandini 2020). In response, individuals increasingly seek to pre-empt such scenarios by asserting control over the timing and manner of their death. Practices such as advance care planning, living wills, and a growing interest in assisted dying exemplify this desire for agency.

In this context, managing death becomes a means of reaffirming autonomy and mitigating the prospect of a frightening and uncertain future. However, this focus on strategic end-of-life planning may risk reinforcing a vicious cycle of ever-greater emphasis on rational control and decision-making. This, in turn, may further impede the capacity to engage with the fundamental uncertainties that inevitably accompany the end of life. For dying remains –by its very nature– unpredictable, uncontrollable, and unmalleable (Rosa 2020, pp. 83–85). The dominant discourse of choice and control may offer an illusion of mastery that fails to address the profound existential adversities people often face when involved during a dying process (Ermers 2025; Sullivan 2002). As previously noted, there is a tangible gap between theoretical control and the actual, lived experience of a lack thereof. A deeper recognition of the anxieties and uncertainties inherent to late modernity (Giddens 1991) may create space for normalising ambivalence as an essential feature of the human condition.

This calls for what I refer to as an ‘ambivalent imagination’. With ambivalent imagination I aim to denote the capacity to envision one’s future in emotionally mixed, conflicting, or uncertain ways, particularly when facing severe illness, decline, or death. It is not merely indecision, but a state in which divergent imagined futures coexist, often in potentially tense or conflicting ways. At the end of life, this may mean that individuals hold multiple futures simultaneously: hope for recovery alongside acceptance of deterioration; the desire for life-prolonging interventions together with a longing for comfort, peace, or closure (Ohnsorge et al. 2012; van Wijngaarden 2024). This ambivalence is not an abstract cognitive exercise. It is embodied, shaped by pain, fatigue, and fear; relational, mediated by families, caregivers, and cultural expectations; and moral, guided by notions of a ‘good death’ and the duties and obligations one carries. Ambivalence thus resides within the self, as the imagination sustains multiple possible identities or outcomes at once, each reflecting significant aspects of identity and generating internal conflict. Rather than signifying a failure of autonomy or mere indecision, ambivalence indicates an attempt to integrate complex realities (van Wijngaarden, 2024). Therefore, ambivalence should be acknowledged, rather than suppressed, in end-of-life decision-making.

Hence there is a need for a more comprehensive vocabulary that extends beyond a predominant lexicon centred around choice, control, preferences and individual autonomy. Instead, in order to reflect the lived complexity of end-of-life experiences, this refined vocabulary should equally incorporate terms like uncertainties, anxieties, dependencies and interdependence, contingency and ambivalence (van Wijngaarden 2024). I propose that this is indispensable to foster more substantive personal and societal dialogues about what it means to live towards the end of life, and ultimately, to die.

## Conclusion

In this article, my goal was to explore how and why choice has become such a prevalent value in Western societies, and how its prominence has significantly shaped the way we approach the end of life. I have also examined the accompanying side effects and the existential challenges that result from this prioritisation of choice and control. A one-sided emphasis on individual autonomy and choice in matters of death and dying fails to acknowledge that these are fundamentally relational and dialogical processes in which choice and control often play only a limited role. Following Taylor, Dodds and Mol, among others, I therefore contend that meaningful engagement with these existential life events requires breaking open the choice-driven discursive frameworks, not only in our private conversations and clinical encounters, but also in the public discourse and academic ethical debates.

A life worth living is not only a life over which we have control, but also a life that takes us by surprise, which overturns our plans. Not only because life remains a precarious affair, but more fundamentally, because we miss something very important when we understand life as a matter of prudence, prevention and control.

(de Wit 2018, p. 165; my translation)
